# Hydrogen Sulfide Inhibits High Glucose-Induced sFlt-1 Production via Decreasing ADAM17 Expression in 3T3-L1 Adipocytes

**DOI:** 10.1155/2017/9501792

**Published:** 2017-06-27

**Authors:** Tian-xiao Hu, Gang Wang, Wei Wu, Lu Gao, Qing-ying Tan, Jing Wang

**Affiliations:** ^1^Department of Endocrinology, Chinese PLA 117th Hospital, Hangzhou 310013, China; ^2^Department of Physiology, Second Military Medical University, Shanghai 200433, China; ^3^Department of Obstetrics and Gynecology, Women's Hospital, School of Medicine, Zhejiang University, Hangzhou 310006, China

## Abstract

Hydrogen sulfide (H_2_S) has recently been identified as an endogenous gaseous signaling molecule. The aim of the present study was to investigate the effect of H_2_S on high glucose- (HG-) induced ADAM17 expression and sFlt-1 production in 3T3-L1 adipocytes. Firstly, we found that HG DMEM upregulated the expression of ADAM17 and production of sFlt-1 in 3T3-L1 adipocytes. Knocking down ADAM17 attenuated the effect of high glucose on sFlt-1 production in adipocytes. HG decreased the expression of CSE and 3-MST, as well as the endogenous H_2_S production. Furthermore, knocking down CSE and 3-MST significantly increased ADAM17 expression and sFlt-1 production. The addition of exogenous H_2_S through the administration of sodium hydrosulfide (NaHS) inhibited HG-induced upregulation of ADAM17 expression and sFlt-1 production. In conclusion, decreased expression of CSE and 3-MST and the subsequent decrease in H_2_S production contribute to high glucose-induced sFlt-1 production via activating ADAM17 in adipocytes. Exogenous H_2_S donor NaHS has a potential therapeutic value for diabetic vascular complications.

## 1. Introduction

Macroangiopathic and microangiopathic complications are major causes of mortality in diabetes. Several studies have demonstrated that elevated circulating soluble vascular endothelial growth factor (VEGF) receptor 1, also called soluble fms-like tyrosine kinase-1 (sFlt-1), is one of the major contributors to the development of macroangiopathic and microangiopathic diseases, such as ischaemic heart disease, chronic kidney disease, and preeclampsia [1–3]. Wieczor et al.'s study has demonstrated that the production of sFlt-1 is significantly upregulated in type 2 diabetes mellitus (T2DM) patients with peripheral arterial disease [4]. In obesity and gestational diabetes mellitus (GDM), adipose tissue, rather than placental tissue, is thought to be the main source of elevated sFlt-1 levels [5]. However, whether or not adipose tissue is the major source of elevated sFlt-1 levels in T2DM patients with peripheral arterial disease remains unknown. Several studies have demonstrated that sFlt-1 can be shedded from the ectodomain of transmembrane Flt-1 by A disintegrin and metalloproteinase 10 (ADAM10) and ADAM17 [6, 7]. In Raikwar et al.'s study [8], overexpression of ADAM17 increasing Flt-1 cleavage while knockdown of ADAM17 reducing Flt-1 cleavage suggested that ADAM17 was responsible for ectodomain shedding of Flt1. Until now, the expression of ADAM17 and release of sFlt-1 in adipocytes exposed to high glucose remain to be elucidated.

Hydrogen sulfide (H_2_S), a lately identified gaseous transmitter, can be produced in a wide spectrum of tissues through the activity of cystathionine-*γ*-lyase (CSE), cystathionine-*β*-synthetase (CBS), and 3-mercaptopyruvate sulfur transferase (3-MST) [9]. Previous studies have shown that H_2_S has anti-inflammatory, antioxidative stress, proangiogenesis, and endothelial protection properties in various tissues [10–12]. In both of adipose tissues and adipocytes, CSE, CBS, and 3-MST are identified [13, 14]. Pan et al. [15] have demonstrated that high glucose inhibits expression of CSE and production of H_2_S in adipocytes. However, whether high glucose affects the expression of another two H_2_S-generating enzymes, CBS and 3-MST, in adipocytes remains unknown. The effects of H_2_S on high glucose-induced aberrant expression of ADAM17 and production of sFlt-1 also need further investigation. In the present study, we hypothesized that H_2_S might be involved in modulation of high glucose-induced ADAM17 expression and sFlt-1 production in adipocytes. To test it, we firstly investigated the expression of ADAM17 and the production of sFlt-1 in 3T3-L1 adipocytes exposed to high glucose. Then, we confirmed the role of ADAM17 in sFlt-1 release in adipocytes using siRNA approach. Furtherly, the effects of H_2_S on high glucose-induced aberrant expression of ADAM17 and increased production of sFlt-1 were investigated.

## 2. Materials and Methods

### 2.1. Cell Culture

3T3-L1 cells (Cell Bank, Shanghai Institutes for Biological Sciences, Chinese Academy of Sciences) were cultured in high-glucose (HG) DMEM (Gibco) containing 10% newborn calf serum (Gibco) at 37°C 5% CO2–95% air. Three days after achieving confluency, cells were incubated in HG DMEM containing 10% (*v*/*v*) fetal bovine serum (FBS) (Gibco), supplemented with 100 milliunits/ml insulin, 0.5 mmol/l 3-isobutyl-1-methylxanthine(Sigma), and 1.0*μ*mol/l dexamethasone (Sigma) for 2 days. The cells were then placed in HG DMEM containing 10% FBS, supplemented with insulin but lacking any other supplements for the additional 2 days to allow 3T3-L1 cells to differentiate into mature adipocytes. The media were replaced every 2 days thereafter until >85% of the cells contained lipid droplets. During the experimental incubation period, 3T3-L1 adipocytes were incubated with low-glucose (LG) DMEM containing 10% (*v*/*v*) FBS.

### 2.2. Drug Treatment

LG DMEM contained 5.5 mmol/l glucose, and HG DMEM contained 25.0 mmol/l glucose. NaHS (Sigma) was dissolved in PBS. TAPI-1 (MCE) was dissolved in dimethyl sulfoxide (DMSO). After 7–10 days of differentiation, 3T3-L1 adipocytes were treated with serum-free LG DMEM or serum-free HG DMEM for 24 h. To determine the effect of H_2_S, cells were treated with serum-free HG DMEM containing NaHS (10*μ*M, 25*μ*M, and 50*μ*M) or serum-free HG DMEM without NaHS for 24 h. To block the function of ADAM17, cells were treated with serum-free HG DMEM containing nonspecific IgG or anti-ADAM-17 monoclonal antibody (D1(A12)) for 24 h. To inhibit the function of ADAM17, cells were treated with serum-free HG DMEM containing DMSO or 20*μ*M TAPI-1.

### 2.3. RNA Interferences

The small interfering RNAs (siRNAs) for CSE, 3-MST, and ADAM17 were designed and synthesized by the GenePharma Corporation (Shanghai, China). The siRNA used in the present study were illustrated in supplements (see Table S1 in Supplementary Material available online at https://doi.org/10.1155/2017/9501792). Control siRNA was in scrambled sequence without any specific target. To knockdown the expression of CSE, 3-MST, and ADAM17, cultured 3T3-L1 adipocytes were transfected with CSE, 3-MST, and ADAM17 siRNAs and negative control (NC) siRNA using Lipofectamine™ 2000 (Invitrogen) for 24 h.

### 2.4. Real-Time H_2_S Production Measurement

Cultured cells were scraped off the plate in the presence of cold RIPA lysis buffer containing protease inhibitor cocktail tablet (Roche, Indianapolis, IN). The lysates were quickly centrifuged at 4°C for 10 minutes, and supernatants were then collected. Then real-time kinetics of H_2_S production was determined by using a miniaturized H_2_S microrespiration sensor (Model H_2_S-MRCh, Unisense, Aarhus, Denmark) coupled to Unisense PA2000 amplifier [16]. Briefly, measurement was performed in a temperature-controlled microrespiration chamber (Unisense). After the sensor signals stabilized, 1.0 mmol/l L-cysteine (Sigma) and 1.0 mmol/l pyridoxal-5′-phosphate (Sigma), a cofactor for the enzymes CBS and CSE, were added. H_2_S production rates were then determined at the initial steepest slopes of each trace.

### 2.5. Western Blot Analysis

Cultured cells were scraped off the plate in the presence of cold radioimmunoprecipitation assay (RIPA) lysis buffer containing protease inhibitor cocktail tablet (Roche, Indianapolis, IN). The lysates were quickly centrifuged at 4°C for 10 minutes. The supernatant was collected and protein concentration was assayed using BCA protein assay kit (Beyotime). 30 *μ*g of protein samples was separated by 10% or 15% SDS-PAGE and subsequently transferred to nitrocellulose membranes. After blockage for 2 hours, membranes were incubated with the antibody against CBS, CSE, 3-MST, ADAM17, and *β*-actin overnight at 4°C. Membranes were then washed and incubated with a secondary horseradish peroxidase- (HRP-) conjugated antibody (Santa Cruz). Immunoreactive proteins were visualized using Immobilon Western Chemiluminescent HRP Substrate (Merck Millipore) and Tanon 5200 Multi scanner. The ratio of band intensities to *β*-actin was obtained to quantify the relative protein expression level.

### 2.6. ELISA Analysis for sFlt-1

Cell-cultured media from different treatment groups were harvested. The contents of sFlt-1 in culture media were determined by ELISA kit (Westang Biotech Co. Ltd., Shanghai, China) according to the manufacturer's instructions. The absorbance was measured at 450 nm wavelength with Denley Dragon Wellscan MK 3 (Thermo).

### 2.7. Statistical Analysis

The data are presented as mean ± SEM. All data were tested for homogeneity of variance by Bartlett's test before analyzing the significance. Individual comparisons were made by one-way ANOVA followed by least significant difference (LSD) *t*-test for the data which were normally distributed. In all of the tests, *P* < 0.05 was considered to be significant.

## 3. Results

### 3.1. High Glucose Upregulates ADAM17 Expression and sFlt-1 Production in 3T3-L1 Adipocytes

**Figure 1:**
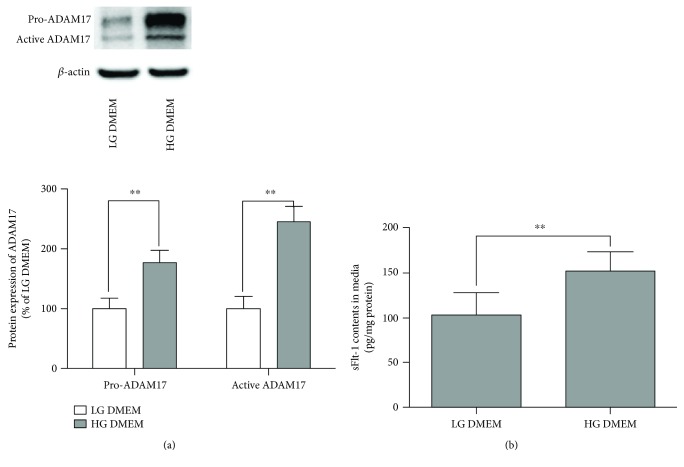
Effects of high glucose on the expression of ADAM17 and production of sFlt-1 in 3T3-L1 adipocytes. 3T3-L1 adipocytes were treated with LG DMEM or HG DMEM for 24 h. (a) Effect of high glucose on the protein expression of pro-ADAM17 and active ADAM17 in 3T3-L1 adipocytes. The protein expression of pro-ADAM17 and active ADAM17 in 3T3-L1 adipocytes was determined by Western blotting as described in Materials and Methods. Representative protein bands were presented on the top of the corresponding histogram. (b) Effect of high glucose on the production of sFlt-1. The production of sFlt-1 in the media was measured by ELISA. Data were presented as mean ± SEM (*n* = 4 cultures). ^∗∗^*P* < 0.01 versus indicated.

ADAM17 has been demonstrated to be responsible for proteolytic process of transmembrane Flt-1 into sFlt-1. In adipocytes treated with LG DMEM and HG DMEM, the protein expression of ADAM17 was determined by Western blotting. As shown in Figure 1(a), the protein expression of both pro-ADAM17 and active ADAM17 significantly increased in 3T3-L1 adipocytes exposed to HG DMEM. sFlt-1 contents in cultured media were determined by ELISA kit. As shown in Figure 1(b), treatment of 3T3-L1 adipocytes with HG DMEM stimulated sFlt-1 production.

### 3.2. High Glucose Stimulates sFlt-1 Production through Increasing ADAM17 Expression in 3T3-L1 Adipocytes

**Figure 2:**
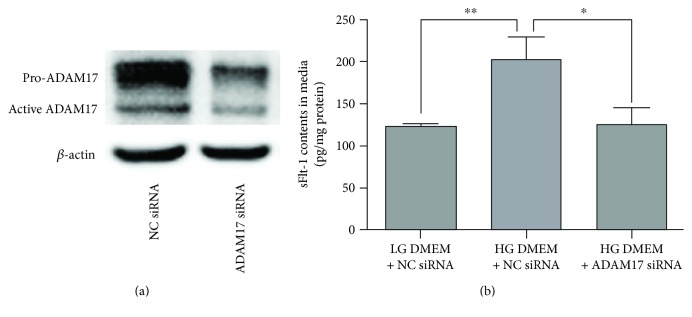
Effects of high glucose on sFlt-1 production in ADAM17 knockdown 3T3-L1 adipocytes. 3T3-L1 adipocytes were transfected with NC siRNA exposed to LG DMEM, NC siRNA exposed to HG DMEM, or ADAM17 siRNA exposed to HG DMEM. (a) Representative protein bands of pro-ADAM17 and active ADAM17 in 3T3-L1 adipocytes transfected with NC siRNA and ADAM17 siRNA exposed to HG DMEM. The protein expression of ADAM17 in 3T3-L1 adipocytes was determined by Western blotting. (b) The effects of high glucose on sFlt-1 production in ADAM17 knockdown 3T3-L1 adipocytes. The production of sFlt-1 in the media was measured by ELISA. Data were presented as mean ± SEM (*n* = 4 cultures). ^∗^*P* < 0.05, ^∗∗^*P* < 0.01 versus indicated.

We then investigated the role of ADAM17 in sFlt-1 release in 3T3-L1 adipocytes exposed to HG DMEM using siRNA approach. Transfection of cells with ADAM17 siRNA caused significant reduction of pro-ADAM17 expression and active ADAM17 expression (Figure 2(a)). As shown in Figure S1, high glucose increased ADAM17 expression in 3T3-L1 adipocytes transfected with NC siRNA and transfection of ADAM17 siRNA decreased ADAM17 expression in 3T3-L1 adipocytes exposed to HG DMEM. As shown in Figure 2(b), high glucose increased sFlt-1 production in 3T3-L1 adipocytes transfected with NC siRNA, and the effects of high glucose on sFlt-1 production were not occurring in 3T3-L1 adipocytes transfected with ADAM17 siRNA.

**Figure 3:**
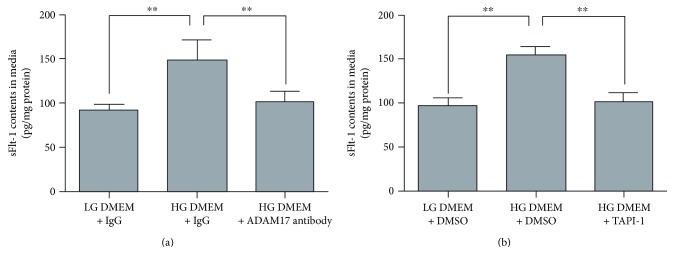
Effects of high glucose on sFlt-1 production in ADAM17-blocked or -inhibited 3T3-L1 adipocytes. (a) The effects of high glucose on sFlt-1 production in ADAM17-blocked 3T3-L1 adipocytes. 3T3-L1 adipocytes were treated with LG DMEM containing nonspecific IgG or HG DMEM containing nonspecific IgG or HG DMEM containing anti-ADAM-17 monoclonal antibody (D1(A12)). (b) The effects of high glucose on sFlt-1 production in ADAM17-inhibited 3T3-L1 adipocytes. 3T3-L1 adipocytes were treated with LG DMEM containing DMSO or HG DMEM containing DMSO or HG DMEM containing TAPI-1. The production of sFlt-1 in the media was measured by ELISA. Data were presented as mean ± SEM (*n* = 4 cultures). ^∗∗^*P* < 0.01 versus indicated.

To further confirm the role of ADAM17 in sFlt-1 production, adipocytes were treated with anti-ADAM-17 monoclonal antibody (D1(A12)) or ADAM17 inhibitor TAPI-1. As shown in Figure 3, the effects of high glucose on sFlt-1 production were not occurring in ADAM17-blocked or -inhibited 3T3-L1 adipocytes.

### 3.3. High Glucose Downregulates CSE and 3-MST Expression, as well as H_2_S Production in 3T3-L1 Adipocytes

**Figure 4:**
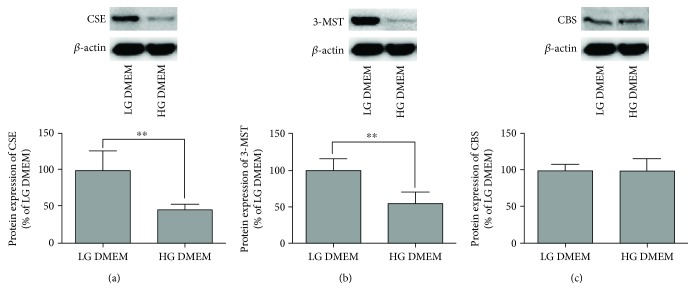
Effects of high glucose on the expression of CSE, 3-MST, and CBS in 3T3-L1 adipocytes. 3T3-L1 adipocytes were treated with LG DMEM or HG DMEM for 24 h, and then, the protein expression of CSE, 3-MST, and CBS was determined by Western blotting as described in Materials and Methods. Upper panels, representative bands for protein expression of CSE, 3-MST, and CBS in 3T3-L1 adipocytes treated with LG DMEM or HG DMEM. Lower panels, the cumulative data of protein expression of CSE, 3-MST, and CBS in 3T3-L1 adipocytes treated with LG DMEM or HG DMEM. Data were presented as mean ± SEM (*n* = 4 cultures). ^∗∗^*P* < 0.01 versus indicated.

It has been reported that high glucose downregulated CSE expression and H_2_S production in adipocytes. In order to confirm the expression of CSE and the production of H_2_S and determine the expression of CBS, 3-MST in adipocytes exposed to high glucose, the protein expression of CBS, CSE, and 3-MST was determined by Western blotting. As shown in Figure 1, HG DMEM downregulated protein expression of CSE (Figure 4(a)) and 3-MST (Figure 4(b)). There was no significant difference in CBS expression between low glucose- and high glucose-treated adipocytes (Figure 4(c)). We also determined real-time H_2_S production in adipocytes exposed to LG DMEM and HG DMEM. As shown in Figure S2, the real-time H_2_S production rate was significantly decreased in adipocyte treated with high glucose.

### 3.4. CSE siRNA and 3-MST siRNA Upregulate ADAM17 Expression and sFlt-1 Production in 3T3-L1 Adipocytes

**Figure 5:**
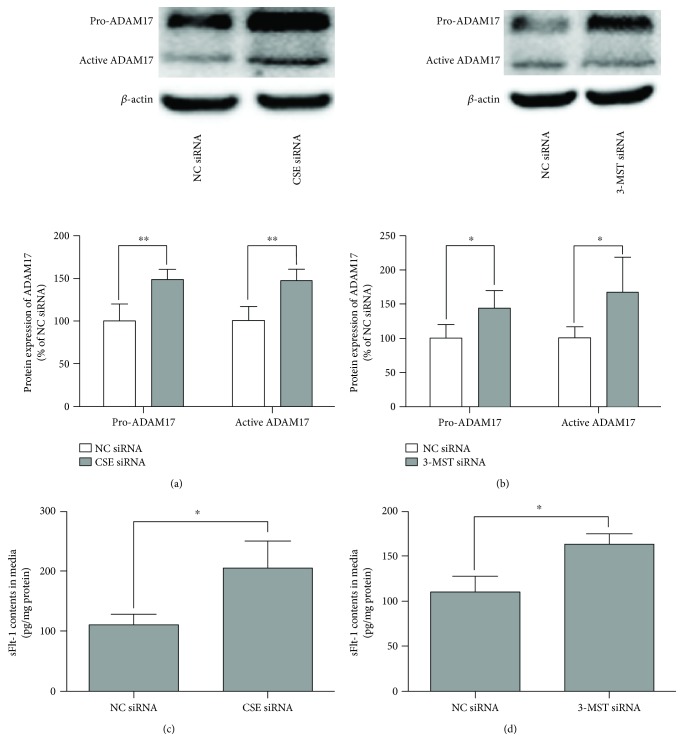
Effects of decreasing CSE and 3-MST expression on the expression of ADAM17 and production of sFlt-1 in 3T3-L1 adipocytes. 3T3-L1 adipocytes were transfected with negative control (NC) siRNA, CSE siRNA, or 3-MST siRNA for 24 h. (a and b) Effect of decreasing CSE (a) and 3-MST (b) expression on the protein expression of pro-ADAM17 and active ADAM17 in 3T3-L1 adipocytes. The protein expression of pro-ADAM17 and active ADAM17 in 3T3-L1 adipocytes was determined by Western blotting as described in Materials and Methods. Representative protein bands were presented on the top of the corresponding histogram. (c and d) Effect of decreasing CSE (c) and 3-MST (d) expression on the production of sFlt-1. The production of sFlt-1 in the media was measured by ELISA. Data were presented as mean ± SEM (*n* = 4 cultures). ^∗^*P* < 0.05, ^∗∗^*P* < 0.01 versus indicated.

We then investigated whether decreasing CSE and 3-MST expression contributes to the effects of high glucose on ADAM17 expression and sFlt-1 production in 3T3-L1 adipocytes by siRNA approach. As shown in Figure S3, transfection of CSE siRNA caused reduction of CSE expression and transfection of 3-MST siRNA caused reduction of 3-MST expression in 3T3-L1 adipocytes exposed to LG DMEM. As shown in Figure 5, CSE siRNA or 3-MST siRNA resulted in increasing the expression of ADAM17 (a-b) and the production of sFlt-1 (c-d) in 3T3-L1 adipocytes exposed to LG DMEM.

### 3.5. NaHS Inhibits High Glucose-Induced Upregulation of ADAM17 Expression and sFlt-1 Production in 3T3-L1 Adipocytes

**Figure 6:**
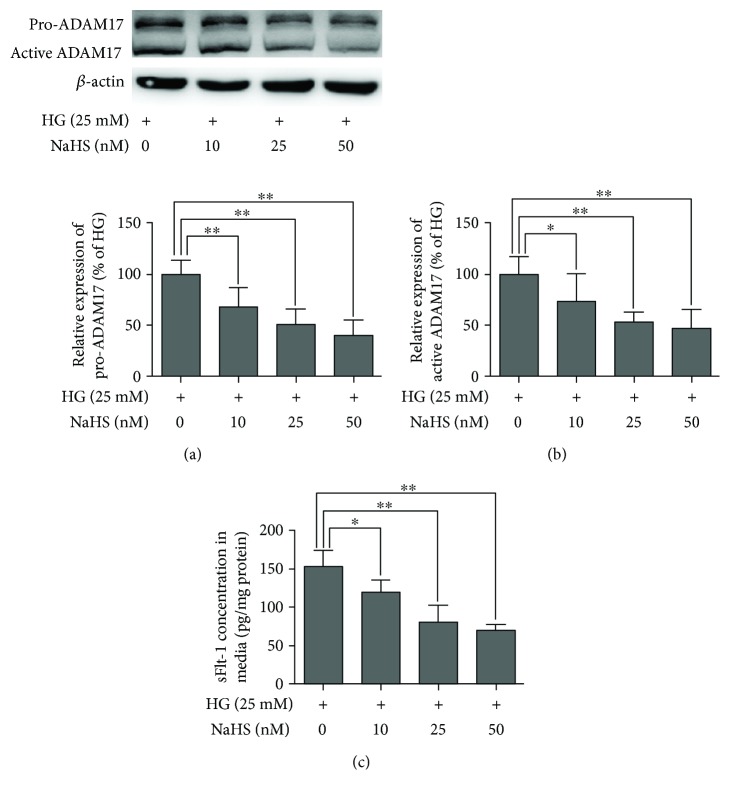
Effects of NaHS on high glucose-induced upregulation of ADAM17 expression and sFlt-1 production in 3T3-L1 adipocytes. 3T3-L1 adipocytes were treated with HG DMEM containing increasing concentration of NaHS for 24 h. (a and b) The effects of NaHS on high glucose-induced upregulation of ADAM17 expression. The protein expression of ADAM17 in 3T3-L1 adipocytes was determined by Western blotting. Representative protein bands were presented on the top of the corresponding histogram. (c) The effects of NaHS on high glucose-induced upregulation of sFlt-1 production in 3T3-L1 adipocytes. The production of sFlt-1 in the media was measured by ELISA. Data were presented as mean ± SEM (*n* = 4 cultures). ^∗^*P* < 0.05, ^∗∗^*P* < 0.01 versus indicated.

In our previous study, we have demonstrated that H_2_S donor NaHS inhibited ADAM10 expression and sFlt-1 release in placental cells. Both ADAM10 and ADAM17 were responsible for ectodomain shedding of Flt1. We found that ADAM17 was expressed in adipocytes whereas ADAM10 was not expressed in adipocytes through PCR assay and Western blotting assay (data for ADAM10 was not shown). In order to confirm the role of H_2_S in high glucose-induced upregulation of ADAM17 expression in 3T3-L1 adipocytes, cultured 3T3-L1 adipocytes were treated with high glucose DMEM containing NaHS for 24 h, a commonly used donor of exogenous H_2_S, and then, pro-ADAM17 and active ADAM17 expression was determined by Western blotting. As shown in Figures 6(a) and 6(b), treatment of 3T3-L1 adipocytes with NaHS (25 and 50 nM) could result in a decrease in pro-ADAM10 and active ADAM10 expression in a dose-dependent manner. sFlt-1 content in cultured supernatant was determined by ELISA kit. As shown in Figure 6(c), treatment of 3T3-L1 adipocytes with NaHS (25 and 50 nM) could result in a decrease in sFlt-1 production in dose-dependent manner.

## 4. Discussion

The present study demonstrated that reduced expression of CSE and 3-MST induced by high glucose significantly stimulated sFlt-1 production via ADAM17 activation in adipocytes. H_2_S donor NaHS suppressed the expression of ADAM17 and the production of sFlt-1 induced by high glucose in adipocytes.

sFlt-1, also called soluble VEGFR-1, is one kind of antiangiogenic factors. When forming complexes with VEGF-A, sFlt-1 decreased biological activity of VEGF-A, thus leading to negative impact on angiogenesis and dysfunction of endothelium. Elevated sFlt-1 was found to be involved in the development of macroangiopathic and microangiopathic diseases since this factor acts as a VEGF antagonist by making them unavailable for signaling to membrane-bound receptors, thereby leading to dysfunction of endothelium [1–3]. Wieczor et al. [4] found that sFlt-1 production in T2DM patients complicated with peripheral arterial disease was higher than that in nondiabetic individuals with peripheral arterial disease. Lappas [5] demonstrated that adipose tissue was a major source of elevated sFlt-1 levels in obesity and GDM. However, sFlt-1 production in adipose tissue of T2DM patients remains to be elucidated. In the present study, we found that high glucose significantly increased sFlt-1 production in adipocytes.

Many soluble proteins are derived from the ectodomain of their transmembrane forms by proteolytic process via specific “sheddases,” such as Matrix metalloproteinases (MMPs) [17, 18] and ADAMs [19–21]. ADAM10 and ADAM17 are two important “sheddases” involved in proteolytic process of transmembrane Flt-1 into sFlt-1 [6–8]. According to our PCR assay and Western blotting assay, ADAM17 was expressed in adipocytes whereas ADAM10 was not expressed in adipocytes (data not shown). ADAM17, also known as TACE (tumor necrosis factor-*α*-converting enzyme), is able to cleave a large variety of substrates including amyloid precursor protein, TNF-*α* [22], Notch [23], and other receptors, as well as many growth factors, cytokines, and cell adhesion molecules [24, 25]. Zhou et al.'s study has demonstrated that transmembrane TNF-*α* was almost completely proteolytically processed into sTNF-*α* by high glucose through activating ADAM17 [26]. This evidence strongly indicated that high glucose activated ADAM17 in adipocytes. In the present study, we showed that high glucose significantly increased ADAM17 expression in adipocytes. Furthermore knocking down ADAM17 abolished the effect of high glucose on sFlt-1 production in adipocytes. In addition, the effects of high glucose on sFlt-1 production were not occurring in ADAM17-blocked or -inhibited 3T3-L1 adipocytes. These results suggest that ADAM17 is involved in the proteolytic process of transmembrane Flt-1 into sFlt-1 induced by high glucose.

Until now, the mechanisms responsible for increased expression of ADAM17 and production of sFlt-1 in adipocytes remain unclear. Our previous study has demonstrated that H_2_S significantly suppresses sFlt-1 release from placental cells and this effect is associated with inhibition of the shedding process of Flt-1 [7]. H_2_S can be produced in a wide spectrum of tissues through the activity of the synthase enzymes including CSE, CBS, and 3-MST [9]. Our results demonstrated that CSE, 3-MST, and CBS were expressed in adipocytes, consistent with the results of previous studies [13, 14]. Both in adipocytes [15] and in human umbilical vein endothelial cells [25], high glucose significantly inhibited the expression of CSE and production of H_2_S. In the present study, we found that high glucose decreased the expression of CSE and 3-MST, as well as the endogenous H_2_S production, but CBS expression was not affected by high glucose. These results indicate that decreasing H_2_S production and CSE, 3-MST expression could stimulate the expression of ADAM17 and the production of sFlt-1 in adipocytes. Based on that, we found that knocking down CSE and 3-MST significantly increased ADAM17 expression and sFlt-1 production in adipocytes. These results suggest that the decreased expression of CSE and 3-MST contributes to elevated ADAM17 expression and sFlt-1 production in adipocytes.

On the basis of the above findings, including the decreased expression of CSE and 3-MST, as well as the endogenous H_2_S production in adipocytes exposed to high glucose, we speculate that the exogenous supply of H_2_S may attenuate high glucose-induced ADAM17 expression and sFlt-1 production. NaHS is a widely used donor of exogenous H_2_S. When dissolved in solution, NaHS rapidly dissociates to Na^+^ and HS^−^. Following this, HS^−^ associates with H^+^ to produce H_2_S. In the present study, our results demonstrated that NaHS, a donor of exogenous H_2_S, significantly inhibited high glucose-induced upregulation of ADAM17 expression and sFlt-1 production in adipocytes. These results suggest that H_2_S has direct effect on ADAM17 expression and sFlt-1 production in adipocytes. It also raises the possibility of the use of NaHS as a potential therapeutic agent for high glucose-induced ADAM17 expression and sFlt-1 production. Although the inhibition effect of high glucose on H_2_S production has been demonstrated in several different types of cells [15, 27, 28], the mechanisms responsible for high glucose on H_2_S synthase remain unclear. It has been reported that high glucose inhibited H_2_S production via TLR4 inflammatory pathway in mouse mesangial cells [28]. In addition, the expression of several microRNAs, including miR-192 [29], miR-204 [30], and miR-217 [31], was upregulated by high glucose. According to bioinformatic analysis on the website of “TargetScan,” the predicted target gene of miR-192 and miR-204 is CSE and the predicted target gene of miR-217 is CBS. These indicate that high glucose may regulate via miR pathway. However, whether TLR4 inflammatory pathway and miR pathway are involved in modulation effect of high glucose on H_2_S synthase needs further investigation. What is more is that the practical effect of H_2_S on high glucose-induced sFlt-1 production and ADAM17 expression in adipose tissue should be investigated in vivo in the future.

## 5. Conclusion

Taken together, our results suggest that H_2_S synthase enzymes CSE and 3-MST play a critical role in modulation of sFlt-1 production and ADAM17 expression in adipocytes. Decreased expression of CSE and 3-MST contributes to high glucose-induced sFlt-1 production via activating ADAM17 in adipocytes. This study also raises the possibility of the use of NaHS as a potential therapeutic agent for diabetic vascular complications. Additional studies are required to confirm these findings in vivo.

## Supplementary Material

Table.S1. The siRNA sequences for CSE, 3-MST, ADAM17. Fig.S1. The effects of high glucose on pro-ADAM17(A) and active-ADAM17(B) expression were not occurred in 3T3-L1 adipocytes transfected with ADAM17-siRNA. The protein expression of ADAM17 in 3T3-L1 adipocytes were determined by western-blotting as described in materials and methods. Data were presented as mean ± SEM (n=3 cultures). ∗P<0.05, ∗∗P<0.01 vs indicated. Fig.S2. The effects of high glucose on real-time H2S production in adipocyte. The real-time H2S production rate was significantly decreased in adipocyte treated with high glucose. The real-time H2S production in adipocyte was determined by using aminiaturized H2S micro-respiration sensor. Fig.S3. Representative protein bands of CSE(A) and 3-MST(B) in 3T3-L1 adipocytes transfected with CSE-siRNA and 3-MST-siRNA. The protein expression of CSE and 3-MST in 3T3-L1 adipocytes were determined by western-blotting as described in materials and methods.
