# Supplementary material for: Hydrogen Sulfide Inhibits High Glucose-Induced sFlt-1 Production via Decreasing ADAM17 Expression in 3T3-L1 Adipocytes
Source: Int J Endocrinol. 2017 Jun 27;2017:9501792. doi: 10.1155/2017/9501792 (PMC5504937; doi:10.1155/2017/9501792)
Supplement: Supplementary file 1 — Table.S1. The siRNA sequences for CSE, 3-MST, ADAM17. Fig.S1. The effects of high glucose on pro-ADAM17(A) and active-ADAM17(B) expression were not occurred in 3T3-L1 adipocytes transfected with ADAM17-siRNA. The protein expression of ADAM17 in 3T3-L1 adipocytes were determined by western-blotting as described in materials and methods. Data were presented as mean ± SEM (n=3 cultures). ∗P<0.05, ∗∗P<0.01 vs indicated. Fig.S2. The effects of high glucose on real-time H2S production in adipocyte. The real-time H2S production rate was significantly decreased in adipocyte treated with high glucose. The real-time H2S production in adipocyte was determined by using aminiaturized H2S micro-respiration sensor. Fig.S3. Representative protein bands of CSE(A) and 3-MST(B) in 3T3-L1 adipocytes transfected with CSE-siRNA and 3-MST-siRNA. The protein expression of CSE and 3-MST in 3T3-L1 adipocytes were determined by western-blotting as described in materials and methods. [file 9501792.f1.docx]

**Supplements**

Table.S1. The siRNA sequences for CSE, 3-MST, ADAM17

| Target gene |  | SiRNA sequence |
| --- | --- | --- |
| CSE | Sense(5′–3′) | CUGCCAUUGACCUGCUAAAtt |
|  | Acti-sense(5′–3′) | UUUAGCAGGUCAAUGGCAGtt |
| 3-MST | Sense(5′–3′) | GCUCAGUAAACAUCCCGUUtt |
|  | Acti-sense(5′–3′) | AACGGGAUGUUUACUGAGCca |
| ADAM17 | Sense(5′–3′) | GGACCAAGGAGGAAAGUAUtt |
|  | Acti-sense(5′–3′) | AUACUUUCCUCCUUGGUCCtt |


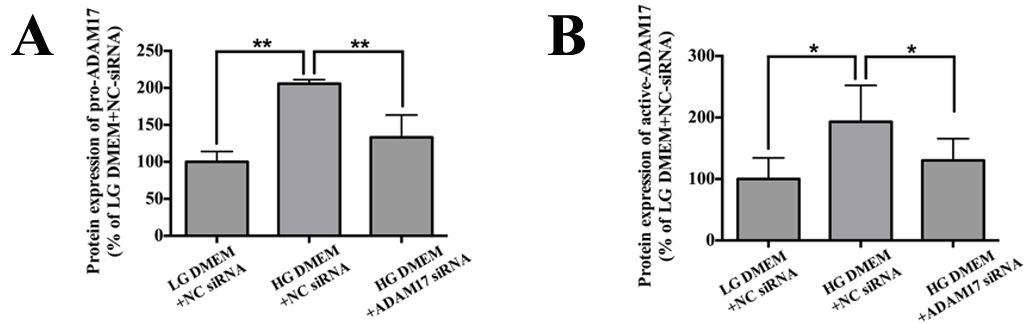
**Fig.S1.** The effects of high glucose on pro-ADAM17(A) and active-ADAM17(B) expression were not occurred in 3T3-L1 adipocytes transfected with ADAM17-siRNA. The protein expression of ADAM17 in 3T3-L1 adipocytes were determined by western-blotting as described in materials and methods. Data were presented as mean ± SEM (n=3 cultures). *P<0.05, **P<0.01 vs indicated.


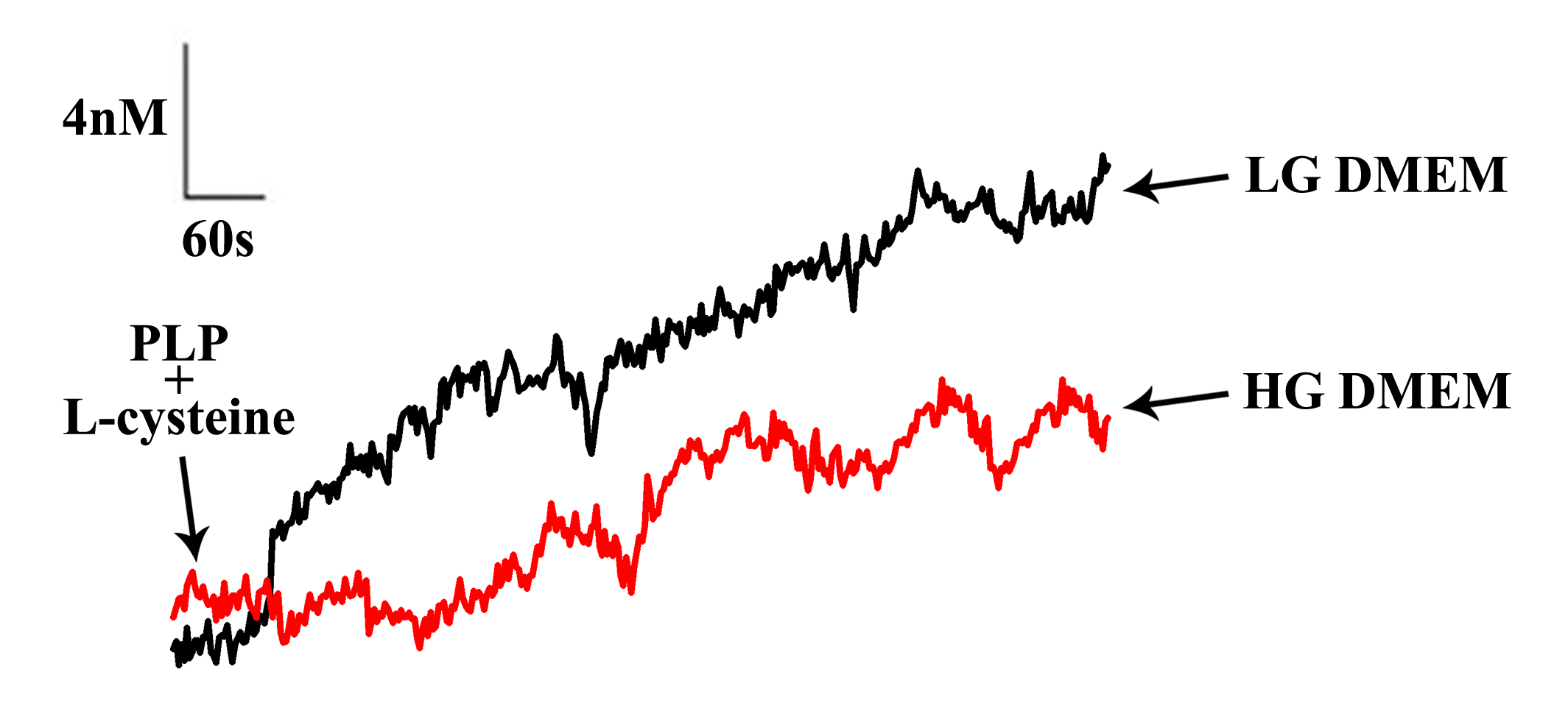


**Fig.S2.** The effects of high glucose on real-time H_2_S production in adipocyte. The real-time H_2_S production rate was significantly decreased in adipocyte treated with high glucose. The real-time H_2_S production in adipocyte was determined by using aminiaturized H_2_S micro-respiration sensor.


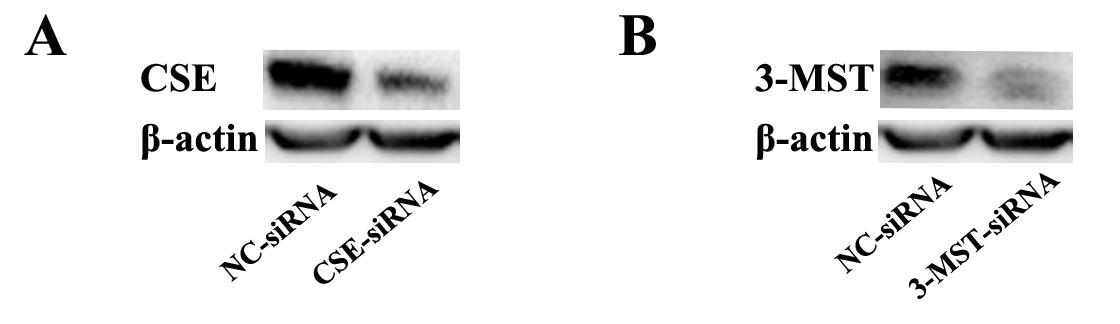


**Fig.S3.** Representative protein bands of CSE(A) and 3-MST(B) in 3T3-L1 adipocytes transfected with CSE-siRNA and 3-MST-siRNA. The protein expression of CSE and 3-MST in 3T3-L1 adipocytes were determined by western-blotting as described in materials and methods.
