# Supplementary material for: Hydrogen Sulfide Inhibits High Glucose-Induced sFlt-1 Production via Decreasing ADAM17 Expression in 3T3-L1 Adipocytes
Source: Int J Endocrinol. 2017 Jun 27;2017:9501792. doi: 10.1155/2017/9501792 (PMC5504937; doi:10.1155/2017/9501792)
Supplement: Supplementary file 2 [file 9501792.f2.pdf]

Supplements

Table.S1. The siRNA sequences for CSE, 3-MST, ADAM17

Target gene		SiRNA sequence
CSE	Sense(5′–3′)	CUGCCAUUGACCUGCUAAAtt
	Acti-sense(5′–3′)	UUUAGCAGGUCAAUGGCAGtt
3-MST	Sense(5′–3′)	GCUCAGUAAACAUCCCGUUtt
	Acti-sense(5′–3′)	AACGGGAUGUUUACUGAGCca
ADAM17	Sense(5′–3′)	GGACCAAGGAGGAAAGUAUtt
	Acti-sense(5′–3′)	AUACUUUCCUCCUUGGUCCtt

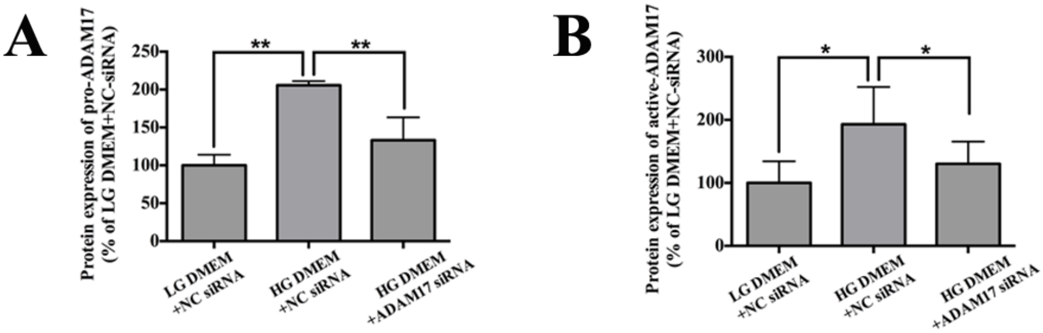

Fig.S1. The effects of high glucose on pro-ADAM17(A) and active-ADAM17(B) expression were not occurred in 3T3-L1 adipocytes transfected with ADAM17-siRNA. The protein expression of ADAM17 in 3T3-L1 adipocytes were determined by western-blotting as described in materials and methods. Data were presented as mean ± SEM (n=3 cultures). *P<0.05, **P<0.01 vs indicated.

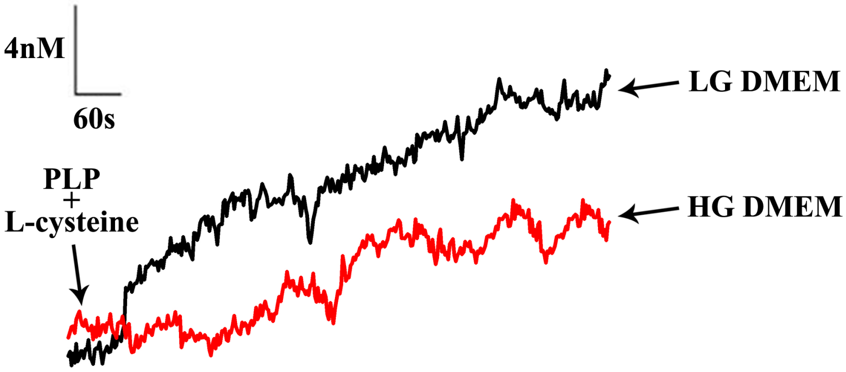

Fig.S2. The effects of high glucose on real-time H_2S production in adipocyte. The real-time H_2S production rate was significantly decreased in adipocyte treated with high glucose. The real-time H_2S production in adipocyte was determined by using aminiaturized H_2S micro-respiration sensor.

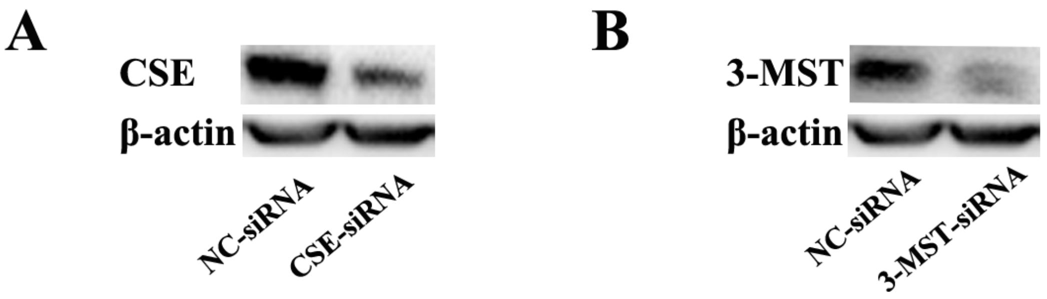

Fig.S3. Representative protein bands of CSE(A) and 3-MST(B) in 3T3-L1 adipocytes transfected with CSE-siRNA and 3-MST-siRNA. The protein expression of CSE and 3-MST in 3T3-L1 adipocytes were determined by western-blotting as described in materials and methods.
